# Molecular surveillance of *Plasmodium falciparum* resistance to sulfadoxine-pyrimethamine among pregnant women attending antenatal clinics in Bobo-Dioulasso, Burkina Faso

**DOI:** 10.1051/parasite/2026035

**Published:** 2026-06-22

**Authors:** Mamoudou Cissé, Alamissa Soulama, Edmond W. Silga, Michel K. Gomgnimbou

**Affiliations:** 1 Laboratoire de recherche, Centre MURAZ Bobo-Dioulasso Burkina Faso; 2 Institut Supérieur des Sciences de la Santé, Université Nazi BONI Bobo-Dioulasso Burkina Faso

**Keywords:** Malaria in pregnancy, Sulfadoxine-pyrimethamine, Molecular markers, Associated factors, Burkina Faso

## Abstract

This study aimed to assess the prevalence and risk factors of molecular markers of *Plasmodium (P.) falciparum* resistance to sulfadoxine-pyrimethamine (SP) among pregnant women in Bobo-Dioulasso, and to examine their association with maternal anaemia. This cross-sectional study was conducted between October and December 2022 among 288 SP-naïve pregnant women attending antenatal care at the Centre Médical Urbain of Lafiabougou. Dried blood spots were collected, and *P. falciparum* infection was detected using quantitative polymerase chain reaction (qPCR) targeting the *varATS* gene. Positive samples were genotyped for mutations in the *Pfdhfr* and *Pfdhps* genes using a nested PCR approach followed by restriction fragment length polymorphism analysis. Multivariable logistic regression models were used to identify predictors of resistance markers and maternal anaemia. Among the 172 qPCR-confirmed *P. falciparum*–positive samples, the *Pfdhfr* triple mutant haplotype (N51I, C59R, S108N) was detected in 33.7% of cases, while the *Pfdhps* A437G mutation was present in 82.7%. No *Pfdhfr* I164L or *Pfdhps* K540E mutations were observed. Gestational age was independently associated with carriage of the triple *Pfdhfr* mutation (adjusted OR = 2.2, 95% CI: 1.1–4.5). Both gestational age (adjusted OR = 2.5, 95% CI: 1.2–4.9) and infection with parasites carrying the triple *Pfdhfr* mutation (adjusted OR = 3.9, 95% CI: 1.8–8.3) were significant predictors of maternal anaemia. The relatively high prevalence of SP resistance markers indicates sustained drug pressure in this setting. Although SP appears to remain effective for intermittent preventive treatment in pregnancy, continued molecular surveillance is warranted to inform malaria control policies.

## Abstract (fr)

Cette étude visait à évaluer la prévalence et les facteurs de risque des marqueurs moléculaires de la résistance de *Plasmodium (P.) falciparum* à la sulfadoxine-pyriméthamine (SP) chez les femmes enceintes de Bobo-Dioulasso, et à examiner leur association avec l'anémie maternelle. Cette étude transversale a été menée entre octobre et décembre 2022 auprès de 288 femmes enceintes n’ayant jamais reçu de SP, suivies en soins prénatals au Centre Médical Urbain de Lafiabougou. Des confettis de sang séché ont été effectués et l'infection à *P. falciparum* a été détectée par PCR quantitative (qPCR) ciblant le gène *varATS*. Les échantillons positifs ont été génotypés pour les mutations des gènes *Pfdhfr* et *Pfdhps* par PCR nichée, suivie d'une analyse du polymorphisme de longueur des fragments de restriction (RFLP). Des modèles de régression logistique multivariée ont été utilisés pour identifier les facteurs prédictifs des marqueurs de résistance et de l'anémie maternelle. Parmi les 172 échantillons positifs à *P. falciparum* confirmés par qPCR, l'haplotype triple mutant *Pfdhfr* (N51I, C59R, S108N) a été détecté dans 33,7 % des cas, tandis que la mutation *Pfdhps* A437G était présente dans 82,7 % des cas. Aucune mutation *Pfdhfr* I164L ni *Pfdhps* K540E n'a été observée. L'âge gestationnel était associé de manière indépendante au portage de la triple mutation *Pfdhfr* (OR ajusté = 2,2 ; IC à 95 % : 1,1–4,5). L’âge gestationnel (OR ajusté = 2,5, IC à 95 % : 1,2–4,9) et l’infection par des parasites porteurs de la triple mutation *Pfdhfr* (OR ajusté = 3,9, IC à 95 % : 1,8–8,3) étaient tous deux des prédicteurs significatifs de l’anémie maternelle. La prévalence relativement élevée des marqueurs de résistance à la sulfadoxine-pyriméthamine (SP) indique une pression médicamenteuse soutenue dans ce contexte. Bien que la SP semble demeurer efficace en traitement préventif intermittent pendant la grossesse, une surveillance moléculaire continue est nécessaire pour éclairer les politiques de lutte contre le paludisme.

## Introduction

Malaria in pregnancy (MiP) remains a major public health concern in sub-Saharan Africa. In 2024, 36% of the 36 million pregnancies recorded in the World Health Organization (WHO) African Region were infected with *Plasmodium* spp. [33]. *Plasmodium (P.) falciparum* is the principal cause of adverse maternal and neonatal outcomes, including maternal anaemia, preterm delivery, stillbirth, low birth weight, and increased maternal and infant mortality [6, 20, 33].

Intermittent preventive treatment with sulfadoxine-pyrimethamine during pregnancy (IPTp-SP) is a cornerstone strategy for reducing the burden of MiP and has demonstrated substantial benefits for both mothers and foetuses [1, 11]. However, its effectiveness is increasingly threatened by the emergence and spread of SP-resistant *P. falciparum* strains [30].

*In vitro* resistance is associated with point mutations in the *P. falciparum* dihydrofolate reductase (*Pfdhfr*) and dihydropteroate synthase (*Pfdhps*) genes [23, 29]. In particular, the quintuple (*Pfdhfr* N51I, C59R, S108N + *Pfdhps* A437G and K540E) and sextuple (*Pfdhfr* N51I, C59R, S108N + *Pfdhps* A437G, K540E, A581G) mutant haplotypes have been strongly associated with reduced IPTp-SP efficacy in sub-Saharan Africa [30]. However, despite reduced antimalarial efficacy in areas of very high resistance, IPTp-SP has been shown to continue reducing the risk of low birthweight and maternal anaemia [30, 34]. Therefore, IPTp-SP remains recommended in such settings until more effective alternatives for malaria chemoprevention become available [30, 34]. These recommendations highlight the importance of continuous molecular surveillance, as early detection of resistance allows timely interventions to limit its spread and impact. Nevertheless, data on the risk factors driving the emergence and dissemination of SP resistance markers among pregnant women remain scarce [3, 16, 21], and the effect of resistant parasite strains on maternal anaemia is still poorly understood [3].

In Burkina Faso, malaria continues to be the leading cause of medical consultations (33.6%), hospitalisations (43.9%), and deaths (8.7%), with approximately 410,549 cases of malaria in pregnancy reported nationwide in 2024 [19]. Sulfadoxine-pyrimethamine was used as a second-line antimalarial treatment before 2005 and has since been adopted for IPTp. Current national guidelines recommend the administration of at least three doses of SP at monthly intervals during pregnancy [18]. Furthermore, since 2014, SP has been used in combination with amodiaquine for seasonal malaria chemoprevention (SMC) in children under five years of age [17]. Despite its widespread and continued use, data on SP resistance among pregnant women in urban settings where SP is still commonly used for self-medication by non-pregnant individuals [22] are limited and outdated. The only available study, conducted in Bobo-Dioulasso city in 2010, reported prevalences of 25.7% for the triple *Pfdhfr* (N51I, C59R, S108N) mutation and 80.2% for the *Pfdhps* A437G mutation [3]. This study aimed to address this knowledge gap by determining the prevalence and associated factors of molecular markers of *P. falciparum* resistance to SP and by assessing the association between resistant parasite strains and maternal anaemia among pregnant women in Bobo-Dioulasso, the second-largest city in Burkina Faso.

## Materials and methods

### Ethical considerations

Ethics approval for this study was obtained from the Comité d'éthique institutionnel de l'Institut pour la Recherche en Sciences de la Santé, Direction régionale de l'Ouest (A-036-2022/CEIRES). Written informed consent was obtained from all participants prior to enrolment. For participants who were unable to read or write, the consent process was conducted in the presence of an impartial witness; in such cases, consent was documented by thumbprint and countersigned by the witness.

### Study design, site, and period

This cross-sectional study was a secondary analysis of data obtained from the HS-RDT-MiP project, which evaluated the performance of an ultrasensitive malaria rapid diagnostic test among pregnant women in Burkina Faso [4]. The study was conducted between October and December 2022 at the maternity unit of the Centre Médical Urbain (CMU) of Lafiabougou, situated in the peri-urban area of Bobo-Dioulasso. The area receives an annual rainfall of approximately 1,000–1,200 mm and experiences intense malaria transmission from May to November. Recent data from the study area reported a prevalence of *P. falciparum* parasitaemia of 19.8% among pregnant women [4]. The facility records an average of 400 antenatal clinic visits per month. In 2024, 68.9% of pregnant women attended at least four antenatal care visits, and coverage of at least three doses of IPTp-SP reached 84.2% [19].

### Study population, data, and sample collection

**Figure 1:**
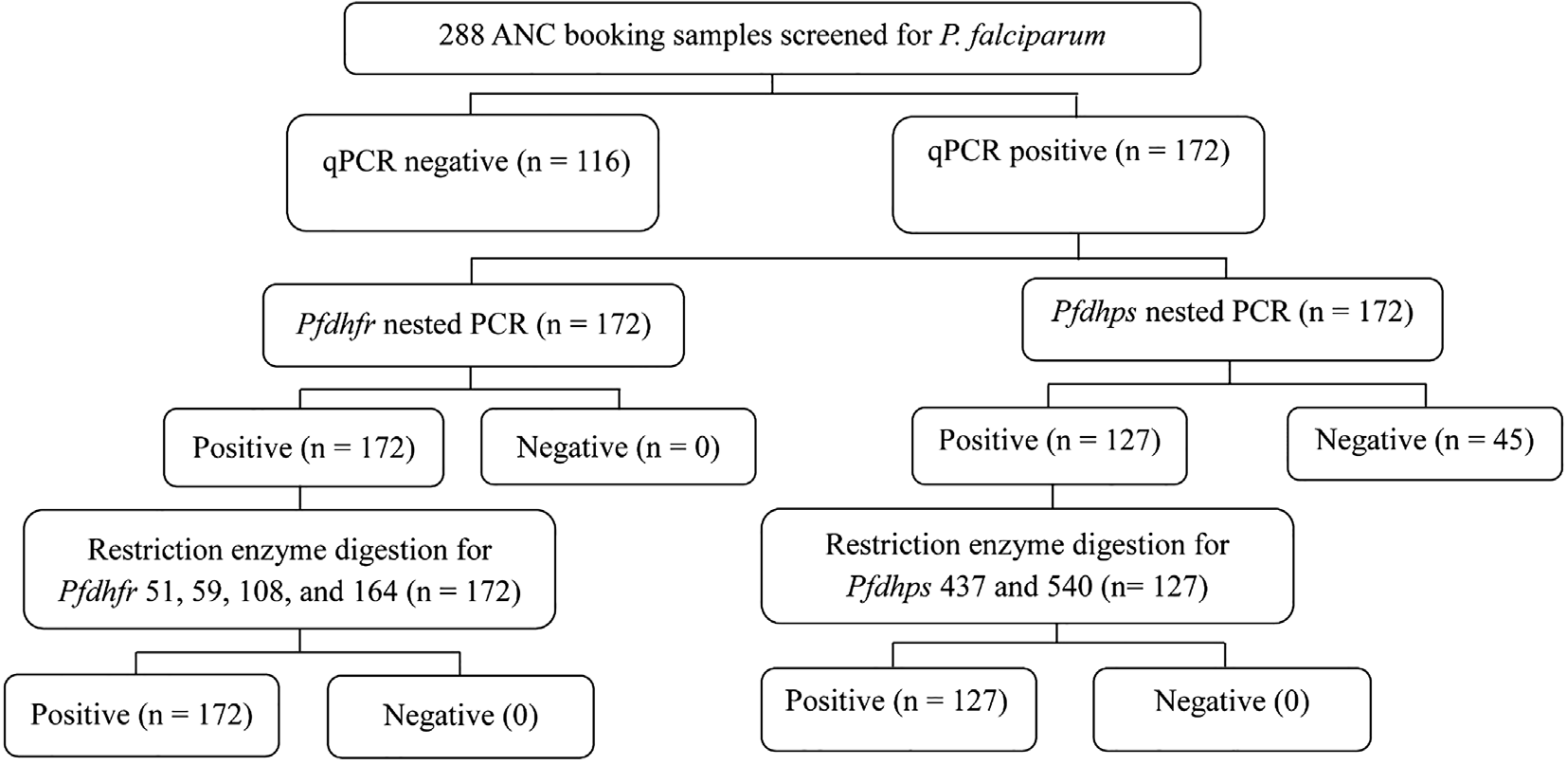
Flowchart of the survey on SP resistance mutations in pregnant women.

In the parent study, 288 pregnant women attending antenatal care at the CMU of Lafiabougou were enrolled [4]. At enrolment, participants were interviewed using a standardised questionnaire to collect sociodemographic characteristics (age, education level) and obstetric information (gravidity, gestational age, and number of antenatal care visits), and body temperature was measured. Venous blood samples were collected from all participants for malaria diagnosis by light microscopy and rapid diagnostic tests, and to ensure sufficient sample volume for multiple laboratory analyses. Dried blood spots (DBS) were prepared on Whatman grade 3 filter paper for molecular analyses because they provide a practical and reliable method for DNA storage and transport under field conditions, particularly in resource-limited settings. The DBS were individually labelled, air-dried at ambient temperature, and stored with silica gel desiccant until analysis. For the present study, molecular genotyping of the *Pfdhfr* and *Pfdhps* genes was performed only on samples from women with confirmed *P. falciparum* infection by quantitative polymerase chain reaction (qPCR) (*n* = 172) (Fig. 1).

### Laboratory procedures

#### Diagnosis of malaria by microscopy

Thick and thin blood smears were prepared and stained with 10% Giemsa for 10 min, with thin smears fixed in methanol prior to staining. Slides were independently examined by two experienced microscopists. Parasite density was determined from thick blood smears by counting asexual parasites against 200 leukocytes, assuming a standard leukocyte count of 8,000 cells/μL of blood [32].

#### DNA extraction and confirmation of *P. falciparum* parasite species by qPCR

*Plasmodium falciparum* DNA was extracted from dried blood spots using QIAamp DNA Mini Kits (QIAGEN, Hilden, Germany), according to the manufacturer’s instructions. DNA was used immediately or stored at −20 °C. Detection was performed by an ultrasensitive qPCR assay targeting the multicopy var gene acidic terminal sequence (varATS) using previously described primers and probe, with minor modifications to the reaction mixture and thermal cycling conditions [12].

#### Genotyping of *Pfdhfr* and *Pfdhps* genes

Single-nucleotide polymorphisms (SNPs) associated with SP resistance were genotyped in the *Pfdhfr* and *Pfdhps* genes using nested PCR followed by allele-specific restriction fragment length polymorphism (RFLP) analysis, as previously described [10]. This method was selected because it is a well-established, reliable, and cost-effective approach for detecting known resistance-associated SNPs in resource-limited settings. The *Pfdhfr* SNPs analysed were N51I, C59R, S108N, and I164L, while *Pfdhps* SNPs included A437G and K540E. Nested PCR products were separated by electrophoresis on 2.5% agarose gels and classified as wild type, mutant, or mixed infections based on band size patterns; mixed infections were considered mutant. The *P. falciparum* Dd2 and 3D7 laboratory clones were used as positive controls and to verify restriction enzyme digestion.

#### Maternal haemoglobin concentration measurement

Haemoglobin concentration was measured using a haemoglobinometer (HemoCue AB, Ängelholm, Sweden). Anaemia was defined as a haemoglobin level <11 g/dL and classified as mild (10.0–10.9 g/dL), moderate (7.0–9.9 g/dL), or severe (<7.0 g/dL), in accordance with WHO criteria [31].

### Data analyses

Data were double-entered using EpiData 3.1, cleaned, and then analysed using STATA version 12.0 (STATA Corporation, College Station, TX, USA).

Descriptive statistics were used to summarise participant characteristics and included frequencies and proportions for categorical variables, and means ± standard deviations or medians with interquartile ranges (IQR) for continuous variables. *Plasmodium falciparum* infection status was classified: a) as negative when both microscopy and qPCR were negative, b) microscopic infection when microscopy was positive, irrespective of qPCR results, and c) submicroscopic infection when microscopy was negative but qPCR was positive. The prevalence of individual *Pfdhfr* mutations (N51I, C59R, S108N, I164L) and *Pfdhps* mutations (A437G, K540E) was estimated, followed by the prevalence of the *Pfdhfr* triple mutant haplotype (N51I, C59R, S108N) and the quadruple mutant haplotype (*Pfdhfr* N51I, C59R, S108N plus *Pfdhps* A437G). Median parasite densities were compared between infections with mutant and wild-type parasites using the Kruskal–Wallis test. Univariate and multivariable logistic regression analyses were conducted to identify factors associated with *Pfdhfr* mutations and the *Pfdhps* A437G mutation. The association between the *Pfdhps* A437G mutation and anaemia was assessed using Pearson’s chi-squared test, while the relationship between the *Pfdhfr* triple mutant haplotype and maternal anaemia was evaluated using univariate and multivariable logistic regression models. Multivariable analyses were performed using backward stepwise selection, with variables retained at *p* < 0.05 and removed at *p* > 0.10. Statistical significance was defined as *p* < 0.05.

## Results

### Prevalence and risk factors for mutations

Molecular genotyping was conducted on 172 *P. falciparum*–positive samples identified by qPCR. Genotyping success rates were 100% (172/172) for the *Pfdhfr* gene and 74% (127/172) for the *Pfdhps* gene (Fig. 1). The baseline characteristics of the study population are presented in Table 1. Participants had a mean age of 25.0 ± 6.6 years, and multigravidae constituted the largest proportion (43.0%), with approximately half being illiterate (51.2%). The median gestational age at enrolment was 16 weeks (IQR: 12–20), and 66.3% of women were enrolled during the second trimester. Most participants were afebrile (96.5%), while anaemia was observed in 60.5%. Submicroscopic *P. falciparum* infection was detected in 67.4% of women, with a geometric mean parasite density of 9.8 parasites/μL (95% CI: 5.7–16.8), as determined by qPCR.

**Table 1:** Characteristics of the study population (*n* = 172).

Variables	Frequency	%
Age (years)		
≤ 20	55	32.0
> 20	117	68.0
Formal schooling		
Yes	84	48.8
No	88	51.2
Gravidity		
Primigravidae	53	30.8
Secundigravidae	45	26.2
Multigravidae	74	43.0
Gestational age (trimester)		
1st trimester	57	33.1
2^nd^ and 3^rd^ trimesters	115	66.9
Number of antenatal clinic visits		
≤ 2	144	83.7
≥ 3	28	16.3
Fever^a,b^		
Yes	6	3.5
No	165	96.5
*P. falciparum* infection		
Microscopic	56	32.6
Submicroscopic	116	67.4
Anaemia		
Yes	104	60.5
No	68	39.5

^a^1 missing data; ^b^Fever was defined as an axillary temperature ≥ 37.5 °C.

**Figure 2:**
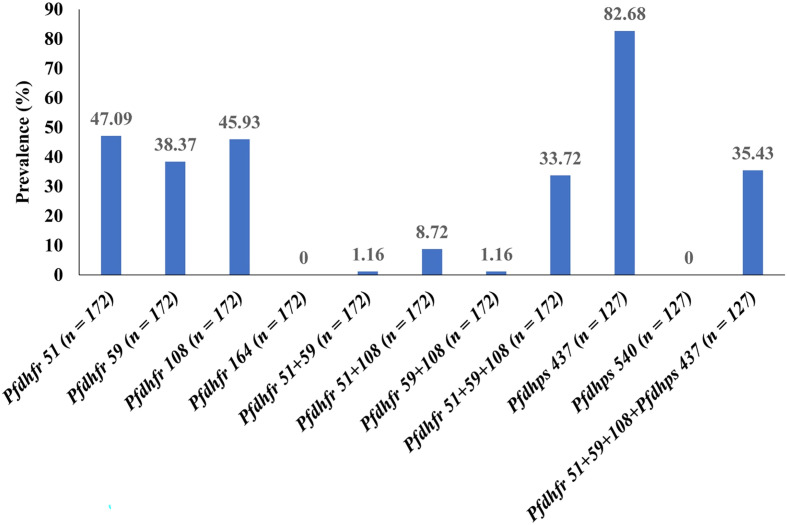
Profile of molecular markers of SP-resistant *P. falciparum* in pregnant women. The sample size (*n*) used to calculate each prevalence is indicated in parentheses on the *x*-axis labels and reflects the number of samples successfully genotyped for the corresponding marker or genotype.

The most prevalent *Pfdhfr* mutation was N51I (47.1%; 81/172; 95% CI: 37.4–58.5), followed by S108N (45.9%; 79/172; 95% CI: 36.4–57.2) and C59R (38.4%; 66/172; 95% CI: 29.7–48.8). No mutations were detected at codon 164 (Fig. 2). The *Pfdhps* A437G mutation was highly prevalent (82.7%; 105/127; 95% CI: 67.6–100.1), whereas the *Pfdhps* K540E mutation was absent. The prevalence of the *Pfdhfr* triple mutant haplotype (N51I, C59R, S108N) was 33.7% (58/172; 95% CI: 25.6–43.6), and that of the quadruple mutant haplotype (*Pfdhfr* N51I, C59R, S108N plus *Pfdhps* A437G) was 35.4% (45/127; 95% CI: 25.9–47.4).

The presence of the *Pfdhfr* N51I, C59R, and S108N mutations, as well as the *Pfdhfr* triple mutant haplotype, was significantly associated with higher parasite density (median ≥ 230 parasites/μL; *p* < 0.001). No such association was observed for the *Pfdhps* A437G mutation (*p* = 0.339) (Supplementary File 1: Table S1).

In univariate analyses, women in the second and third trimesters had approximately twice the odds of carrying the *Pfdhfr* N51I mutation (crude OR = 2.1; 95% CI: 1.1–4.0) (Table 2) and the *Pfdhfr* triple mutant haplotype (crude OR = 2.2; 95% CI: 1.1–4.5) (Table 3) compared with women in the first trimester. These associations remained significant in multivariable analyses for both the *Pfdhfr* N51I mutation (adjusted OR = 2.1; 95% CI: 1.1–4.0) and the *Pfdhfr* triple mutant haplotype (adjusted OR = 2.2; 95% CI: 1.1–4.5). None of the variables examined were significantly associated with the *Pfdhps* A437G mutation (Supplementary File 2: Table S2).

**Table 2:** Risk factors for single mutations in the *Pfdhfr* gene.

Variable	*Pfdhfr* N51I mutation			*Pfdhfr* C59R mutation			*Pfdhfr* S108N mutation		
	cOR^a^ (95% CI)	aOR^b^ (95% CI)	*p*	cOR^a^ (95% CI)	aOR^b^ (95% CI)	*p*	cOR^a^ (95% CI)	aOR^b^ (95% CI)	*p*
Age (years)									
≤ 20	0.9 (0.5–1.7)	–	–	0.9 (0.5–1.7)	–	–	1.3 (0.7–2.6)	–	–
> 20	1	–	–	1	–	–	1	–	–
Gravidity									
Paucigravidae	1.1 (0.6–2.0)	1.2 (0.6–2.1)	0.642	0.9 (0.8–2.7)	1.0 (0.5–1.8)	0.961	1.5 (0.8–2.7)	1.6 (0.8–2.9)	0.163
Multigravidae	1	1	–	1	1	–	1	1	–
Gestational age									
1st trimester	1	1		1	1		1	1	
2nd and 3rd trimesters	**2.1 (1.1**–**4.0)**	**2.1 (1.1**–**4.0)**	**0.028**	1.7 (0.9–3.4)	1.7 (0.9–3.4)	0.109	1.7 (0.9–3.3)	1.7 (0.9–3.5)	0.072

^a^Crude odd ratio; ^b^Adjusted odd ratio.

**Table 3:** Risk factors for the triple *Pfdhfr* mutation

Variable	Triple *Pfdhfr* mutation			
	cOR^a^ (95% CI)	*p*	aOR^b^ (95% CI)	*p*
Age (years)				
≤ 20	1.1 (0.5–2.1)	0.875	–	–
> 20	1	–	–	–
Gravidity				
Paucigravidae	1.1 (0.6–2.1)	0.756	1.2 (0.6–2.3)	0.613
Multigravidae	1	–	1	–
Gestational age				
1st trimester	1	–	1	–
2nd and 3rd trimesters	**2.2 (1.1**–**4.5)**	**0.035**	**2.2 (1.1**–**4.5)**	**0.035**

^a^Crude odds ratio; ^b^Adjusted odds ratio.

### Association between mutations and anaemia

In univariable analyses, second and third trimester pregnancy (crude OR = 2.8, 95% CI: 1.5–5.4), microscopic malaria infection (crude OR = 4.0, 95% CI: 1.9–8.4), and infection with parasites carrying the *Pfdhfr* triple mutant haplotype (crude OR = 4.3, 95% CI: 2.0–9.1) were associated with increased odds of maternal anaemia (Table 4). In multivariable analyses, gestational age (adjusted OR = 2.5, 95% CI: 1.2–4.9) and the *Pfdhfr* triple mutant haplotype (adjusted OR = 3.9, 95% CI: 1.8–8.3) remained independently associated with anaemia (Table 4).

**Table 4:** Risk factors for maternal anaemia

Variable	Anaemia			
	cOR^a^ (95% CI)	*p*	aOR^b^ (95% CI)	*p*
Age (years)				
≤ 20	1.2 (0.6–2.4)	0.560	–	–
> 20	1	–	–	–
Gravidity				
Paucigravidae	1.3 (0.7–2.4)	0.388	–	–
Multigravidae	1	–	–	–
Gestational age				
1st trimester	1	–	1	–
2nd and 3rd trimesters	**2.8 (1.5**–**5.4)**	**0.002**	**2.5 (1.2**–**4.9)**	**0.010**
Formal schooling				
No	1.2 (0.7–2.3)	0.491	–	–
Yes	1	–	–	–
*P. falciparum* infection				
Microscopic	**4.0 (1.9**–**8.4)**	**< 0.001**	–	–
Submicroscopic	1	–	–	–
Triple *Pfdhfr* mutation				
No	1	–	1	–
Yes	**4.3 (2.0**–**9.1)**	**< 0.001**	**3.9 (1.8**–**8.2)**	**0.001**

^a^Crude odds ratio; ^b^Adjusted odds ratio.

Anaemia prevalence was lower among women infected with parasites carrying the *Pfdhps* A437G mutation (62.9%) than among those without the mutation (72.7%), although this difference was not statistically significant (*p* = 0.77).

## Discussion

Monitoring molecular markers of *P. falciparum* resistance to SP is essential for evaluating the continued effectiveness of IPTp-SP and guiding malaria control policies during pregnancy. This study analysed both microscopic and submicroscopic *P. falciparum* infections among SP-naïve pregnant women, 12 years after IPTp-SP implementation in Burkina Faso.

A high prevalence of submicroscopic *P. falciparum* infections (67.4%) was observed in the study. This finding is of major public health concern because submicroscopic infections are often asymptomatic and undetectable by routine microscopy, yet they may constitute an important reservoir sustaining malaria transmission. In pregnant women, these low-density infections may also contribute to adverse maternal and foetal outcomes. The high prevalence of submicroscopic carriage observed here underscores the limitations of microscopy-based surveillance alone and highlights the need for more sensitive diagnostic and surveillance strategies to support malaria control and elimination efforts.

Against this background of persistent parasite circulation, the prevalence of the *Pfdhfr* triple mutant haplotype (N51I, C59R, S108N) increased from 19.2% in Bobo-Dioulasso in 2010 [3] to 32.7% in the present study, indicating a substantial rise over time. A comparable increase has been reported in rural Burkina Faso, where prevalence among pregnant women receiving IPTp-SP rose from 11.4% in 2010 to 63.3% in 2015 [25]. This upward trend likely reflects sustained drug pressure resulting from the continued use of SP for IPTp, self-medication in the general population, and its deployment in SMC [24, 25]. Consistent with this, recent data showed high prevalence of *Pfdhfr* N51I (87%) and near fixation of *Pfdhfr* C59R and S108N mutations among children receiving SMC in Burkina Faso [24]. Although the prevalence observed in this study was lower than that reported in earlier surveys among SP-naïve pregnant women in rural Burkina Faso (44.9%) [5], those findings date back to an earlier period (January 2010–December 2011), and resistance patterns may have changed over time. Our estimates were also lower than those reported among first-antenatal-clinic attendees in Ghana who had not received SP (81%) [9], a setting characterized by higher malaria transmission intensity. However, the Ghanaian estimates may have been affected by a lower genotyping success rate (73.7%) compared with the complete genotyping achieved in the present study.

Consistent with recent reports from Burkina Faso [24, 26, 28, 35], none of the *P. falciparum* isolates analysed in this study carried the *Pfdhfr* I164L mutation. This mutation, which is associated with high-level SP resistance [13], remains absent in most West African settings [5, 9], except Senegal, where a prevalence of 11.7% has been reported among pregnant women [7]. Higher prevalences have been documented in Central and East Africa, including the Republic of Congo (1.6%) [8] and Uganda (36%) [2].

In contrast, a high prevalence of the *Pfdhps* A437G mutation (82.7%) was observed in the present study, in agreement with previous findings from Burkina Faso [24, 25, 28] and other African countries [2, 5]. In the same study area, this mutation was reported at a lower prevalence (78.8%) in 2010 among SP-naïve pregnant women [3], indicating a gradual increase over time. As A437G is typically the first mutation to emerge in the *Pfdhps* gene [29], its increasing prevalence may represent an early indicator of further selection of mutations at codons 540, 581, and 613, which are associated with higher levels of SP resistance. The higher prevalence observed in this study compared with reports from Ghana among SP-naïve pregnant women (32.2%) [9] may reflect differences in antifolate drug pressure, including the widespread use of co-trimoxazole, which may confer cross-resistance to sulfadoxine [14].

The absence of the *Pfdhps* K540E mutation is consistent with previous reports from Bobo-Dioulasso among pregnant women [3] and children [24], although low-level circulation has been reported in rural areas of Burkina Faso [25, 28]. Given that more than 30% of infections carried the quadruple mutant haplotype, continued molecular surveillance remains essential. Although reduced IPTp-SP efficacy has been reported in regions with high prevalence of quintuple and sextuple mutations, particularly in East and Southern Africa, protective effects against maternal anaemia and low birth weight persist [30]. Overall, the absence of the key resistance-associated mutations *pfdhps* K540E and *pfdhfr* I164L in this setting supports the continued effectiveness and use of SP for IPTp, while highlighting the importance of ongoing surveillance of resistance markers.

In the present study, women in the second and third trimesters of pregnancy were at increased risk of infection with *P. falciparum* parasites carrying the *Pfdhfr* triple mutant haplotype compared with women in the first trimester. Although cumulative exposure to SP during pregnancy could theoretically explain this association, this is unlikely given that only SP-naïve women were included. Moreover, pregnancy is known to be associated with increased susceptibility to infection with multiple *P. falciparum* clones, with the multiplicity of infection (MOI) increasing across trimesters [15, 27]. This may have led to an overestimation of the prevalence of the triple mutant haplotype if individual mutations were present in different clones within the same infection. As the MOI was not assessed in the current study, future investigations should evaluate SP resistance markers alongside *P. falciparum* MOI.

Importantly, the *Pfdhfr* triple mutant haplotype was significantly associated with maternal anaemia among *P. falciparum*-infected pregnant women, even after adjustment for gestational age. One possible explanation is that these mutations were associated with higher parasite densities in the present study, thereby increasing haemolysis and contributing to anaemia. However, this finding contrasts with a previous study conducted in the same setting, which reported no association between resistance markers, parasite density, and maternal anaemia [3]. These inconsistent findings warrant further studies to better elucidate the relationship between SP resistance, parasite burden, and pregnancy-related anaemia.

In this study, women in the second and third trimesters of pregnancy were at increased risk of anaemia. This finding may be partly explained by the higher *P. falciparum* parasite densities observed in these women. To our knowledge, this type of association has not previously been reported among pregnant women infected with *P. falciparum*. These results highlight the need for enhanced surveillance and targeted interventions for anaemia among pregnant women in later stages of pregnancy, particularly those with malaria infection. Strengthening early detection and prompt management of malaria, together with closer monitoring of haemoglobin levels during the second and third trimesters, may help reduce the burden of malaria-associated anaemia.

This study has some limitations. Participants were recruited from a single public health facility in Bobo-Dioulasso, which may limit the generalizability of the findings. In addition, *Pfdhps* genotyping was unsuccessful for 26% of samples, possibly due to the lower sensitivity of nested PCR compared with qPCR or poor DNA quality. Third, other relevant SP resistance markers, including *Pfdhps* I431V, S436A, A581G, and A613S, were not assessed because of financial and laboratory resource constraints. Although the selected markers represent the most widely validated indicators of SP resistance relevant to IPTp-SP efficacy in West Africa, inclusion of additional mutations could have provided a more comprehensive assessment of SP resistance patterns. Future studies should therefore incorporate a broader panel of molecular markers to strengthen surveillance of antifolate resistance dynamics.

## Conclusion

The prevalence of the *Pfdhfr* triple mutant haplotype in Bobo-Dioulasso was relatively high and has increased since 2010, along with the prevalence of the *Pfdhps* A437G mutation. No *Pfdhfr* I164L or *Pfdhps* K540E mutations were detected. Gestational age was significantly associated with the presence of the *Pfdhfr* triple mutant haplotype. Among *P. falciparum*-infected pregnant women, gestational age and infection with parasites carrying the *Pfdhfr* triple mutant haplotype were the main predictors of maternal anaemia. Overall, these findings indicate that SP remains effective in this setting, while underscoring the need for continued surveillance of SP resistance markers, including *Pfdhps* A581G, in conjunction with assessments of multiplicity of infection.

## Supplementary material

*Table S1*: Relationship between parasite density and mutations in the *Pfdhfr* and *Pfdhps* genes. The data showed an association between Plasmodium falciparum density and the following mutations: *Pfdhfr* N51I, *Pfdhfr* C59R, *Pfdhfr* S108N, triple *Pfdhfr*, and *Pfdhps* A437G. This association was determined using the Kruskal–Wallis test. *Table S2*: Risk factors for the *Pfdhps* A437G mutation. The data provided showed the factors associated with the *Pfdhps* A437G mutation. This was done using univariate and multivariable logistic regression analysis. The supplementary material of this article is available at https://www.parasite-journal.org/10.1051/parasite/2026035/olm.
