# Supplementary material for: Molecular surveillance of Plasmodium falciparum resistance to sulfadoxine-pyrimethamine among pregnant women attending antenatal clinics in Bobo-Dioulasso, Burkina Faso
Source: Parasite. 2026 Jun 22;33:36. doi: 10.1051/parasite/2026035 (PMC13290108; doi:10.1051/parasite/2026035)
Supplement: Supplementary file 1 — Table S1: Relationship between parasite density and mutations in the Pfdhfr and Pfdhps genes. The data showed an association between Plasmodium falciparum density and the following mutations: Pfdhfr N51I, Pfdhfr C59R, Pfdhfr S108N, triple Pfdhfr, and Pfdhps A437G. This association was determined using the Kruskal–Wallis test. [file parasite-33-36-s1.pdf]

Table S1. Relationship between mutations in the *Pfdhfr* and *Pfdhps* genes and parasite density

Mutation	Median of parasite density (IQR*)		*p*
	**No**	**Yes**	
Pfdhfr N51I mutation	0.4 (0.2-1.5)	230.8 (38.4-821.2)	< **0.001**
Pfdhfr C59R mutation	0.6 (0.3-6.1)	341.2 (50.2-1420.3)	< **0.001**
Pfdhfr S108N mutation	0.4 (0.2-1.6)	234.6 (43.8-1031.9)	< **0.001**
Triple *Pfdhfr* mutation	0.7 (0.3-7.4)	420.8 (125.2-1505.1)	< **0.001**
Pfdhps A437G mutation	98.1 (14.4-676.6)	38.4 (1.5-409.7)	0.339

IQR: Interquartile range
