# Supplementary material for: Molecular surveillance of Plasmodium falciparum resistance to sulfadoxine-pyrimethamine among pregnant women attending antenatal clinics in Bobo-Dioulasso, Burkina Faso
Source: Parasite. 2026 Jun 22;33:36. doi: 10.1051/parasite/2026035 (PMC13290108; doi:10.1051/parasite/2026035)
Supplement: Supplementary file 2 — Table S2: Risk factors for the Pfdhps A437G mutation. The data provided showed the factors associated with the Pfdhps A437G mutation. This was done using univariate and multivariable logistic regression analysis. [file parasite-33-36-s2.pdf]

Table S2. Risk factors for *Pfdhps* A437G mutation

Variable	*Pfdhps* A437G mutation			
	cOR[a] (95% CI)	***P***	aOR[b] (95% CI)	***p***
**Age (years)**				
≤ 20	0.5 (0.2-1.2)	0.101	0.7 (0.2-2.0)	0.486
> 20	1	-	1	-
**Gravidity**				
Paucigravidae	0.4 (0.1-1.1)	0.063	0.4 (0.1-1.1)	0.063
Multigravidae	1	-	1	-
**Gestational age**				
1st trimester	1	-	-	-
2nd and 3rd trimester	1.2 (0.4-3.2)	0.761	-	-
